# Current Therapy and Short-Term Results of Open Abdominal Management Among Non-trauma Patients

**DOI:** 10.7759/cureus.113840

**Published:** 2026-08-03

**Authors:** Mayu Kitamoto, Kazunori Nojiri, Yukari Takata, Shohei Maruta, Yohei Ota, Hirokazu Suwa, Hidetaka Ono, Takafumi Kumamoto

**Affiliations:** 1 Surgery, Yokosuka Kyosai Hospital, Yokosuka, JPN

**Keywords:** acute care surgery, damage control surgery, negative wound pressure, non-trauma patients, open abdominal management

## Abstract

Introduction

Damage control surgery (DCS) with open abdominal management (OAM) has been established for the treatment of trauma patients; however, in recent years, it has been increasingly expanded to include the management of non-trauma patients with severe conditions. Since 2020, we have performed OAM combined with negative pressure wound therapy (NPWT) for critically ill non-trauma patients.

Methods

We retrospectively reviewed 43 patients who underwent OAM between April 2020 and March 2024. The primary outcome was in-hospital mortality, and the secondary outcomes were postoperative complications, primary fascial closure rate, and duration of OAM. Analyses of postoperative complications, duration of OAM, and primary fascial closure were restricted to patients who survived beyond 24 hours, because these outcomes could not be meaningfully assessed in patients who died shortly after the initial surgery.

Results

Forty-three patients (67% were men) were included in the primary outcome. The median (interquartile range) age was 71 (59-80) years, and the median Charlson Comorbidity Index was 5 (range, 4-7). Indications for OAM included bowel perforation (52%), peritonitis due to strangulated bowel obstruction (16%), postoperative anastomotic leakage (12%), intra-abdominal hemorrhage (9%), non-occlusive mesenteric ischemia (9%), and superior mesenteric artery thrombosis (2%). The mortality rate was 18.6%. Three patients who died within 24 hours were excluded from the secondary outcome analyses, leaving 40 patients for evaluation. The complication rate was 58%, and 23% of cases were classified as Clavien-Dindo grade ≥III. Intra-abdominal complications occurred, including anastomotic leakage, intra-abdominal abscess, and intestinal ischemia. Moreover, pulmonary and vascular complications (e.g., pneumonia, cerebral infarction, and ischemic heart disease) were observed. Primary fascial closure was achieved in all 40 patients included in the secondary outcome analysis (100%).

Conclusion

OAM with NPWT for non-trauma patients may be considered an option for patients with severe general conditions or when intra-abdominal reevaluation is necessary. Further studies are required to improve mortality and complication rates.

## Introduction

Damage control surgery (DCS) with open abdominal management (OAM) is an established technique in patients with severe trauma [1,2]. This technique has been used in patients with major vascular or visceral injuries [3,4]. In recent years, it has been increasingly adapted and expanded to include the management of non-trauma patients with severe conditions [1,5]. Indications for OAM in non-trauma patients include multiple conditions such as peritonitis, mesenteric ischemia, gastrointestinal hemorrhage, and pancreatitis [1,2,5].

In patients with hemodynamic instability, OAM allows abbreviated surgery, facilitates subsequent reassessment and source control, when necessary, permits delayed intestinal reconstruction after adequate resuscitation, and helps prevent intra-abdominal hypertension and abdominal compartment syndrome (ACS) caused by visceral edema [5,6]. After definitive resuscitation and hemodynamic stability are achieved, anatomical reconstruction and abdominal closure can be performed [6,7].

Open abdominal management (OAM), in which the skin and fascia are intentionally left unclosed after laparotomy, requires temporary abdominal closure (TAC). Various TAC techniques have been described, and negative pressure wound therapy (NPWT) has been associated with delayed primary fascial closure rates of approximately 90% [6].

Although OAM is increasingly used in non-trauma patients, available data regarding outcomes in this population remain limited [1,5]. We conducted a retrospective case series to evaluate short-term outcomes of non-trauma patients who underwent OAM. The primary outcome was in-hospital mortality, and the secondary outcomes were postoperative complications, primary fascial closure rate, and duration of OAM.

## Materials and methods

This retrospective case series included consecutive non-trauma patients who underwent OAM at our hospital between April 2020 and March 2024.

OAM was performed based on the World Society for Emergency Surgery (WSES) guidelines [8]. At our institution, the indications for OAM were as follows: (1) severe hemodynamic instability (including septic shock) requiring vasopressor support, in which abbreviated surgery was necessary; (2) the need for planned reassessment of intestinal blood flow or bleeding control when persistent hemodynamic instability, questionable bowel viability, or uncontrolled intra-abdominal contamination precluded primary fascial closure; and (3) inability to achieve primary fascial closure because of severe bowel edema with concern for abdominal compartment syndrome (ACS). OAM was not performed solely because of bowel perforation but only when one or more of the above criteria were present. A planned second-look operation was performed 24-48 hours later according to the patient's clinical condition. During each re-exploration, intestinal blood flow, bowel edema, and the degree of intra-abdominal contamination were assessed to determine whether to continue OAM or proceed with additional bowel resection, intestinal anastomosis, ostomy construction, or primary fascial closure. This process was repeated at short intervals until definitive abdominal closure was considered feasible. At the end of OAM treatment, the fascia was closed with interrupted absorbable sutures. In all patients following the index operation, temporary abdominal closure was achieved using the AbThera® Dressing Kit (3M+KCI, San Antonio, TX, USA) with intermittent negative pressure of 125 mmHg until definitive fascial closure. Patients remained intubated throughout the OAM period and were extubated once definitive fascial closure had been achieved and hemodynamic stability was confirmed.

In patients who developed surgical site infection (SSI), the wound was treated with open wound care, daily wound cleansing, and repeated debridement. When severe wound contamination persisted despite these measures, NPWT using a vacuum-assisted closure (VAC) system was initiated, applying intermittent negative pressure (75 mmHg).

Septic shock was defined by the Third International Consensus Definitions for Sepsis and Septic Shock (Sepsis-3) published in 2016 [9]. Patients with septic shock were identified by persistent hypotension requiring vasopressors to maintain a mean arterial pressure of ≥65 mmHg and a serum lactate level of >2 mmol/L (18 mg/dL) despite adequate fluid resuscitation [9].

The aim of this study was to describe the clinical characteristics and evaluate the short-term outcomes of non-trauma patients who underwent OAM. Patient characteristics included the following factors: age, sex, comorbidities, Charlson Comorbidity Index (CCI) [10], body mass index (BMI), preoperative mean arterial pressure (MAP), and quick sequential organ failure assessment (qSOFA) score [9]. qSOFA was selected because it reflects the physiological condition at the time the decision to perform OAM was made.

The primary outcome was in-hospital mortality and was evaluated in all patients. The secondary outcomes included postoperative complications, primary fascial closure rate, and duration of OAM. Only patients who survived more than 24 hours after OAM initiation were included in the analysis of secondary outcomes, because OAM-related outcomes, including primary fascial closure and postoperative complications, could not be meaningfully assessed in patients who died within 24 hours after the initial surgery. Postoperative complications were defined as adverse events occurring after surgery that were not present preoperatively or indicated worsening of preexisting conditions. Complications were graded according to the Clavien-Dindo classification [11]. Primary fascial closure was defined as the successful approximation and closure of the abdominal fascia following OAM.

Continuous variables were expressed as median and interquartile range (IQR). Categorical variables were expressed as numbers and percentages.

## Results

A total of 43 consecutive patients underwent OAM during the study period. The patient characteristics are summarized in Table 1. The median age of the patients was 70 years (IQR: 59-81 years), and 65% of the patients were men. Approximately 70% of the patients had at least one comorbidity, and the median CCI score was 5 (IQR, 4-7). The median MAP on hospital arrival was 118 mmHg (IQR, 92-142 mmHg), and the median qSOFA score was 2 (IQR, 1-3).

**Table 1:** Patient characteristics Continuous variables were expressed as medians (interquartile ranges). Categorical variables are presented as numbers (%). CCI: Charlson Comorbidity Index; MAP: mean arterial pressure; qSOFA: Quick sequential organ failure assessment.

	n=43
Age, years	71 (59-80)
Male sex	29 (67%)
BMI	22 (18-24)
Comorbidity	31 (72%)
CCI	5 (4-7)
Preoperative MAP	118 (92-142)
qSOFA score	2 (1-3)

The underlying diseases requiring OAM are presented in Table 2. Bowel perforation was the most common indication (52%), followed by strangulated bowel obstruction (16%) and postoperative anastomotic leakage (12%). The other patients presented with intraperitoneal hemorrhage (9%), non-occlusive mesenteric ischemia (9%), and superior mesenteric artery thrombosis (2%). Among patients with bowel perforation, colorectal perforation was the most frequent etiology.

**Table 2:** Underlying diseases requiring OAM OAM: Open abdominal management.

Underlying diseases requiring OAM	n (%)
Bowel perforation (upper gastrointestinal perforation, n=2; small bowel perforation, n=4; colorectal perforation, n=16)	22 (52%)
Strangulated bowel obstruction	7 (16%)
Postoperative anastomotic leakage	5 (12%)
Intraperitoneal hemorrhage	4 (9%)
Non-occlusive mesenteric ischemia	4 (9%)
Superior mesenteric artery thrombosis	1 (2%)

The in-hospital mortality is summarized in Table 3. For the primary outcome, the overall in-hospital mortality rate was 18.6% (8/43). Three patients (6.9%) died before completion of OAM, all within 24 hours after the initial surgery, and all deaths were attributed to sepsis. Five (11.6%) patients died after completion of OAM, including four who died within 30 days after the initial surgery. The causes of death were multi-organ failure due to sepsis (2/5), acute respiratory distress syndrome (1/5), pneumonia (1/5), and cerebral infarction (1/5).

**Table 3:** In-hospital mortality OAM: Open abdominal management.

	n=43
Overall	8 (18.6%)
Before OAM completed	3 (6.9%)
After OAM completed	5 (11.6%)

In-hospital mortality was calculated for all patients, whereas analyses of postoperative complications, primary fascial closure, and OAM duration were restricted to patients surviving beyond 24 hours. Three patients died within 24 hours after the initial surgery and were excluded from the secondary outcome because these outcomes could not be meaningfully assessed in patients who died shortly after the initial surgery. The remaining 40 patients were included in the analysis. The secondary outcomes are summarized in Table 4. The overall postoperative complication rate was 58% (23/40). Nine patients developed complications that were classified as Clavien-Dindo grade ≥III. The median duration of OAM was two days (IQR, 2-4). Primary fascial closure was achieved in all 40 patients included in the secondary outcome analysis (100%).

**Table 4:** Postoperative outcomes OAM: Open abdominal management.

	n=40
Postoperative complications	23 (58%)
Clavien–Dindo grade ≥III	9 (23%)
Primary fascial closure	40 (100%)
OAM duration, days	2 (2–4)

Complications are summarized in detail in Table 5. The most common complications were surgical site infection (SSI), pneumonia, anastomotic leakage, and intra-abdominal abscess. None of the patients developed enteroatmospheric fistula (EAF). Three patients developed SSI, all of which were classified as Clavien-Dindo grade I. Two patients developed superficial incisional SSI on postoperative days 3 and 8, respectively, and one developed deep incisional SSI on postoperative day 10. Because these infections did not improve with open wound care, daily wound cleansing, and repeated debridement, NPWT was initiated in all three patients.

**Table 5:** Complications in detail Some patients experienced more than one complication.

Complication	n (%)
Surgical site infection (SSI) (superficial incisional SSI, n=2; deep incisional SSI, n=1)	3 (13%)
Pneumonia	3 (13%)
Anastomotic leakage	3 (13%)
Intra-abdominal abscess	3 (13%)
Ischemic bowel	1 (4.3%)
Dysphagia	2 (8.7%)
Ventilator dependence	2 (8.7%)
Bowel obstruction	1 (4.3%)
Cerebral infarction	1 (4.3%)
Acute myocardial infarction	1 (4.3%)
Acute respiratory distress syndrome	1 (4.3%)
Multiorgan failure due to sepsis	2 (8.7%)
Enteroatmospheric fistula	0 (0%)

## Discussion

OAM was initially established in patients with trauma to prevent or reduce the severity of the trauma triad of death, including hypothermia, acidosis, and coagulopathy [12,13]. In previous studies, the indications for OAM differed between patients with trauma and without trauma. OAM was performed in non-trauma patients for the following indications: severe intra-abdominal infection, ACS, hemodynamic instability, and intestinal ischemia [4,8,14]. In non-trauma patients, the indication for OAM is often less well established than that in trauma settings. Non-trauma patients frequently include elderly individuals with multiple comorbidities and severe sepsis [8,15-17], which complicates intraoperative decision-making. In such situations, surgeons should carefully consider the need for rapid source control against the risks of prolonged operative time and physiological deterioration. Since 2020, at our hospital, we have been actively performing OAM in patients with non-traumatic diseases who are critically ill or require re-evaluation of intestinal blood flow and bleeding control. In our study, bowel perforation was the most frequent underlying pathology; however, OAM was not selected because of the perforation alone. Rather, OAM was reserved for patients with severe physiological derangement requiring abbreviated surgery, marked bowel edema preventing tension-free fascial closure, persistent hemodynamic instability, questionable bowel viability, or uncontrolled intra-abdominal contamination requiring planned re-exploration. These characteristics distinguish our cohort from patients undergoing a simple planned relaparotomy after definitive source control.

Subramanian et al. [15] reported that elderly patients who underwent OAM had significantly more comorbidities with regard to cardiovascular, pulmonary, and renal diseases. Bruns et al. [16] reported that the median age of non-trauma patients who underwent OAM was 61 years and the median CCI was 2. Considering these reports, non-trauma patients who underwent OAM had various comorbidities. Although the median age in our study was consistent with that previously reported, the median CCI in our study was relatively higher.

It was reported that the mortality rate of non-trauma patients who underwent OAM ranges from 19% to 66.6% [2,3,6,8,16,18-20]. Kritayakirana et al. [21] reported that the open abdominal technique for DCS is associated with a significantly higher mortality rate (31%) among non-trauma patients requiring emergency surgery. The mortality rate observed in our series is consistent with the previously published literature. Because patients who died within 24 hours after the initial operation could not undergo definitive fascial closure or develop postoperative complications, these patients were excluded only from the secondary outcome analyses. Nevertheless, this analytical approach may have resulted in selection bias, because the reported primary fascial closure rate and postoperative complication profile reflect outcomes only among patients who survived the initial postoperative period. Therefore, these secondary outcomes should be interpreted with caution.

Previous studies have reported that the complication rate of non-trauma patients who underwent OAM ranged from 43.2% to 94.9% [1,3,6,8]. The most common complications after OAM are respiratory failure and SSI [5]. According to the reported analysis, irrespective of age, 16%-20% of non-trauma patients who underwent OAM developed postoperative respiratory failure, and 24%-27% developed wound complications [1,3,16]. The complication rate observed in our series was consistent with that reported in previously published studies, and most complications required only medical management. Although SSI was the most common complication in our study, all patients were successfully managed with NPWT alone, and no additional surgery was required.

Miller et al. reported that delayed fascial closure beyond eight days was associated with progressively increased complications and mortality [22]. Prolonged OAM results in lateral retraction of the abdominal wall, making primary fascial closure increasingly difficult [22,23]. Therefore, current guidelines recommend early fascial closure whenever physiologically feasible [23,24]. In the present study, among patients who survived beyond the initial postoperative period, the median duration of OAM was only two days, and primary fascial closure was achieved early in all evaluable patients. This early closure strategy may have contributed to avoiding complications associated with prolonged OAM, although the retrospective design precludes causal inference. NPWT is widely used as a temporary abdominal closure technique because it protects the abdominal contents, removes contaminated fluid, reduces edema, and minimizes fascial edge retraction [25,26]. In our cohort, NPWT was applied in all patients during OAM, and primary fascial closure was achieved after a median OAM duration of two days. Although these findings are encouraging, the absence of a control group prevents any conclusion regarding the independent effect of NPWT on fascial closure. EAF is a serious complication of prolonged OAM [27,28]. No patients in our series developed EAF. However, because the median duration of OAM was only two days, this finding is likely attributable to early abdominal closure rather than NPWT itself. Therefore, the present study cannot determine whether NPWT independently reduced the risk of EAF.

From a clinical perspective, the present study suggests that OAM can be safely applied in carefully selected non-trauma patients requiring abbreviated laparotomy or planned reassessment. High rates of early primary fascial closure were achieved in this cohort managed using NPWT; however, because this was a single-arm retrospective study without a control group, the specific contribution of NPWT cannot be determined. Careful patient selection and reassessment are essential to minimize complications and improve outcomes.

This study had several limitations. First, it was a retrospective, single-center study, and was therefore subject to potential selection bias. Second, the small sample size may limit the generalizability of our findings to other centers. Third, all patients underwent temporary abdominal closure using NPWT, and no comparison group managed with an alternative temporary abdominal closure technique was available. Therefore, the independent effect of NPWT on outcomes, including primary fascial closure and prevention of EAF, could not be determined. Furthermore, three patients who died within 24 hours after the initial surgery were excluded from the analyses of postoperative complications, duration of OAM, and primary fascial closure because these outcomes could not be meaningfully assessed. Although in-hospital mortality was analyzed in all consecutive patients, restricting the secondary outcome analyses to patients surviving beyond 24 hours may have introduced selection bias and may have led to overestimation of the primary fascial closure rate and underestimation of postoperative morbidity. Therefore, these findings should be interpreted cautiously.

## Conclusions

Our findings suggest that OAM with NPWT is feasible for the treatment of non-trauma patients when rapid source control, physiological stabilization, and planned re-exploration are required. Nevertheless, further studies are needed to optimize patient selection and improve the mortality and complication rates.
